# On an Equal Distribution of Excitement, and of the Circulating Fluids

**Published:** 1822-11

**Authors:** 


Art. IV.—*On an equal Distribution of Excitement, and of the Circulating Fluids.* By Dr. Kinglake.

The functions of both animal life and health are performed by a given degree of excitement, by which the circulating fluids are duly distributed to every part of the frame, whence result the stimulus of vascular distention, and a regular supply of all the materials necessary to the various secretions of the animal system. When the excitable power is so equally acted on as to produce a degree of excitement suited to the healthful condition of every part of the frame, the most natural and perfect state of vital action inevitably ensues. The constituent principles entering into the composition of every part of the animal body possess, in their separate and combined properties, the power of sustaining a vital union with every other portion of the frame. It is, therefore, owing to the partial and universal connexion of this power, that excitement or vital action is either regularly or irregularly diffused over the system.

The effects of excitants acting on the excitability vary so much with circumstances that it cannot be defined what may be the precise influence of occurring agents,—whether they may operate equably and generally, so as to induce salutary excitement, or in a more partial and limited manner, so as to occasion local disease. The natural and healthful causes of excitement secure an equal distribution of salutary action over the frame. Wholesome nutriment, pure air, moderate exercise, suitable temperature, and necessary evacuations, tend to diffuse most congenially the kind and degree of excitement that are most natural and healthy over the whole system. The direct effect of this equable excitement is to extend a regular and proportionate transmission of the circulating fluids to every part of the frame.

From this unimpeded circulation arises the indispensable excitement proceeding from a natural degree of vascular distention, and by which materials are supplied to all the various secreting processes of life, whether referring to nervous power, or to the principle of animation,—to the production of temperature,—to the several secreted fluids more directly formed by the gastric, enteric, hepatic, renal, testicular, ovarial, and membranous textures,—or to the nutritive and restorative functions incessantly going on for the sustenance and reparation of the decompositions and wastes of animal existence. An equal distribution both of excitement and of the circulating fluids is of the utmost moment to health, and can only be obtained and preserved by the beneficial influence of salutary agents.

Excitement, or vital action, like the circulating fluids, may be variously diffused and accumulated. If it obtain disproportionately in any particular part, in that situation, active disease may arise, whilst in other portions of the frame deficient excitement may exist, which would be shown in torpor and debility. When the equable state of natural excitement over the whole system is disturbed, those parts which may chance at the time to be most excitable, or rather those which from organic structure are most readily impressed, will become disproportionately affected. In these situations disease will arise, differing in character and intensity according to the texture and uses of the part in which the predominant excitement may show itself. If it be on the brain, or any other visceral organ, the occurring symptoms will characterize the nature and extent of the affection. Connected with this unequal distribution or exaltation of vital action, will be an additional afflux of fluids, which, by unduly distending the loaded vessels, will greatly augment the subsisting excitement. The vascular fulness resulting from this accumulation of excitement and fluidity will soon disable the transmitting powers, so as to induce congestive plenitude or a gorged state of vessel, in which the circulation is retarded almost to stagnation. In parts which are not visceral, such as the muscular, ligamentous, tendinous, cellular, membraneous, or osseous textures, immoderate excitement, aggravated by vascular distention and obstruction, would be a serious ailment, and if not speedily remedied must endanger the integrity of the structure in which it may have occurred. Adhesions, accretions, suppurations, or gangrenous decomposition of structure, must inevitably result from an unrelieved continuance of active disease of this complicated description. On a vital organ, especially the brain, where inordinate excitement and sanguineous determination are most apt to occur, such morbid excitement and accumulation could not fail to endanger life. The lungs, liver, and intestines, are also prone to become affected by an undue distribution of excitement and of the circulating fluids, but in these situations the symptoms are not so violent nor so directly threatening to life. The excitability of the animal system is the creature or effect of organization, and is, in its distributive property, capable of being variously and extensively transferred, according to natural and acquired habits of association or sympathetic influence. The sudden transfers to which excitement is liable appear to be arbitrary, but they are no doubt depending on established laws, which render such occurrences, in the circumstances in which they happen, absolutely and inevitably necessary. The immediate cause of those abrupt shiftings in the seat of morbid excitement lies concealed amongst those arcana of nature that appertain to the minute and recondite operations of material power. The possibility, however, of such an occurrence is a circumstance of practical importance, and legitimately belongs to the physiological history of morbid excitement. Counter-irritation is employed in medical practice as a remedy to effect the removal of active disease from one part to another. It would seem that the excitability can only be stimulated to a given extent, and that, if it be intensely excited in one situation, it must be at the expence of depriving other parts of it, and more particularly those in which it may exuberantly abound. Whether increased excitement take place from associated influence or from mechanically instituting it, an equal diversion may be effected from the part suffering under a high degree of morbid irritation.

An unequal diffusion of excitement, and an undue distribution of the circulating fluids, may mutually occasion each other, and in turn be the cause and effect of each other's influence. Neither state can long exist without involving the other as an inevitable effect. The two conditions are inseparably associated in the close relation of cause and effect. Diseased excitement cannot arise without causing an additional flow of the circulating fluids to the unduly excited part, nor can a redundancy of fluids in any portion of the frame happen without farther stimulating the containing vessels. The conjoint influence of these two states, morbid excitement and vascular distention, though arising in the series of cause and effect, affords a condition of disease afflicting in any situation, but necessarily attended with great danger of life when befalling a vital organ. These diseased states are not referable to the system generally, but rather to certain portions in which the healthy tone and harmony of life have undergone some change. Immoderate excitement, and a superabundant flow of circulating fluids throughout the whole frame, cannot simultaneously occur: such a state would be too universal to be admitted by circumstances that necessarily give to such conditions partial agency. The ailments, therefore, that flow from an unequal distribution of excitement and of the circulating fluids have a local origin, and it will be found, by due attention, that the occurring symptoms are sufficiently demonstrative of the seat of the disease. The general affection induced by the sympathizing participation of the whole system with the morbid part is no proof of original disease, nor should it divert the curative indication from its correct endeavour to relieve the part affected, as the only possible means of subduing the efficient cause of the prevailing malady. An affection of the brain, as well as of the digestive organs, will often draw the whole system into severe and almost undistinguishable ailment; but the extended character of the disease is sympathetic only,—is the effect of a local cause, nor can any curative benefit be afforded without remedying the morbid circumstances by which the affected part is disordered.

The forms of disease resulting from an unequal distribution of excitement and of the circulating fluids may be as various as constitutional differences and temperamental idiosyncracies can admit. The latitude of definite and indefinite affection, under the influence of such powerful causes, is wide enough to include in its spacious range a vast diversity of ailments. It would appear to furnish an adequate cause for every description of inflammatory and febrile excitement. In defining what is meant by inflammatory excitement, it may be said to refer to those conditions of disease in which vital action is so augmented as to threaten disorganization or decomposition of the affected part. In such circumstances, the vital properties of the diseased part,—such as the excitable, the sensorial, the associative, and the functional,—are undergoing hurtful, perhaps destructive, changes. Those morbid conflicts might arise in every part of the animal frame, and the character of the resulting affection will best evince the nature and degree of the existing disease. Febrile excitement differs, perhaps, only from that which is denominated inflammatory, in having had a more obscure origin, in having arisen from a larger but less active sphere of morbid surface than the more insulated and inflammatory description of disease. Visceral derangement, whether owing to inordinate excitement, to redundant fluids, to disturbed or vitiated secretions, or to eventual sympathies or associative consent with other parts, may gradually and imperceptibly disseminate over the whole frame a diseased influence, shown in variable temperature, and in an excessive and deficient exertion of vital power. This febrile or general character of disease, though originating in a portion of the system, is likely to endure as long as the cause remains, or until the system, either from exhausted power or acquired familiarity with the exciting cause, shall cease to partake in the suffering of the disordered part. The various noxious agents that operate on the excitability so as to induce disease act by occasioning an inordinate excitement, and a consequent unequal distribution of the circulating fluids. Through those morbid conditions, specific effects, characteristic of the occurring ailment, may be produced. It is by an unequal distribution of excitement and of the circulating fluids that diseases may be said to locate themselves in the system, and at length to generate the various affections that result from the agency of particular causes.

When no undue excitement prevails and the circulating fluids are equally distributed, the most natural and harmonious state of health may be supposed to exist; but either excess or deficiency in those salutary conditions of vital action must induce diseases differing in their nature and symptoms, but equally deserving notice and relief. The immoderate excitement attending vascular distention occasions much immediate suffering and tends irreparably to disorganize the part subjected to such morbid violence. In the situation in which neither sufficient excitement nor the necessary vascular distention could find access, owing to the obstruction produced by an undue accumulation of those states in other parts, symptoms of deficient energy amounting to torpor and inaction will arise. If an excessive quantity of the circulating fluids be sent to the brain, much hurtful distention and obstruction would be induced in that organ, at once preventing a regular flow of fluids through it, and diminishing by the pressure of its detained contents the nervous power, which has its chief origin in the cerebral structure. Either the liver or the lungs may be morbidly oppressed by having a larger proportion of the circulating fluids cast on them than they can transmit, the result of which will be serious disease; whilst other parts of the frame may be sinking from deficient excitement, for want of a more equal distribution of the circulating fluids.

[To be continued.*]

* We regret that we are under the necessity of dividing Dr. KINGLAKE'S paper, from the great length of some of the ORIGINAL COMMUNICATIONS, inserted before we received the proof sent to him to Taunton.—EDITORS.

